# Anti-FcαRI Monoclonal Antibodies Resolve IgA Autoantibody-Mediated Disease

**DOI:** 10.3389/fimmu.2022.732977

**Published:** 2022-03-15

**Authors:** Amelie Bos, Esil Aleyd, Lydia P. E. van der Steen, P. J. Winter, Niels Heemskerk, Stephan M. Pouw, Louis Boon, Rene J. P. Musters, Jantine E. Bakema, Cassian Sitaru, Michel Cogné, Marjolein van Egmond

**Affiliations:** ^1^ Department of Molecular Cell Biology and Immunology, Amsterdam UMC, Vrije Universiteit, Research Institute of Amsterdam Institute for Infection and Immunity, Research Institute of Amsterdam Institute for Infection and Immunity, Amsterdam, Netherlands; ^2^ Reseach and Development, JJP Biologics, Warsaw, Poland; ^3^ Department of Physiology, Amsterdam UMC, Vrije Universiteit, Amsterdam, Netherlands; ^4^ Department of Otolaryngology/Head-Neck Surgery, Amsterdam UMC, Vrije Universiteit, Amsterdam, Netherlands; ^5^ Department of Dermatology, University of Freiburg, Freiburg, Germany; ^6^ Department of Immunology, University of Limoges, Limoges, France; ^7^ Department of Surgery, Amsterdam UMC, Vrije Universiteit, Amsterdam, Netherlands

**Keywords:** FcαRI, CD89, IgA, neutrophils, LABD

## Abstract

Immunoglobulin A (IgA) is generally considered as a non-inflammatory regulator of mucosal immunity, and its importance in diversifying the gut microbiota is increasingly appreciated. IgA autoantibodies have been found in several autoimmune or chronic inflammatory diseases, but their role in pathophysiology is ill-understood. IgA can interact with the Fc receptor FcαRI on immune cells. We now established a novel IgA autoimmune blistering model, which closely resembles the human disease linear IgA bullous disease (LABD) by using genetically modified mice that produce human IgA and express human FcαRI. Intravital microscopy demonstrated that presence of IgA anti-collagen XVII, - the auto-antigen in LABD-, resulted in neutrophil activation and extravasation from blood vessels into skin tissue. Continued exposure to anti-collagen XVII IgA led to massive neutrophil accumulation, severe tissue damage and blister formation. Importantly, treatment with anti-FcαRI monoclonal antibodies not only prevented disease, but was also able to resolve existing inflammation and tissue damage. Collectively, our data reveal a novel role of neutrophil FcαRI in IgA autoantibody-mediated disease and identify FcαRI as promising new therapeutic target to resolve chronic inflammation and tissue damage.

## Introduction

Immunoglobulin A (IgA) is the dominant antibody subclass present in mucosal areas and plays an important role in the mucosal immune system (1, 2). A delicate balance between tolerating harmless antigens and commensals versus maintaining robust protection against pathogens is necessary to maintain homeostasis. IgA is produced as dimeric molecule (dIgA) by local plasma cells in the lamina propria and released into the lumen as secretory IgA (SIgA) (1). SIgA mainly serves as an antiseptic coating at the mucosa by, amongst others, neutralizing bacterial toxins as well as preventing adherence and invasion of microorganisms. Additionally, it has recently become clear that mucosal IgA plays an important role in diversifying the gut microbiota and community networks, which promotes symbiosis (3–5).

IgA is generally considered as a non-inflammatory antibody. SIgA, which is present in the lumen of mucosal areas, has indeed poor opsonic capacity due to (partial) blockage of the binding site for the IgA Fc receptor FcαRI by secretory component (6). The role of systemic IgA is not completely understood. It is the second most prevalent antibody in serum (after IgG), and has a dual function through interaction with FcαRI. It was demonstrated that binding of monomeric serum IgA to FcαRI induces inhibitory signals *via* immunoreceptor tyrosine-based activation motif (ITAM), which is expressed in the FcR γ chain that associates with FcαRI. This is referred to as ITAMi signaling and as such it was proposed that FcαRI has an anti-inflammatory role under physiological conditions (7, 8). In contrast, crosslinking of FcαRI by IgA immune complexes activates FcαRI-expressing neutrophils, monocytes and CD103^+^ dendritic cells, resulting in pro-inflammatory responses (9–12).

IgA autoantibodies, increased IgA or aberrant IgA immune complexes are found in several diseases, such as celiac disease (13, 14), IgA nephropathy (15–17), IgA vasculitis (18), rheumatoid arthritis (19), multiple sclerosis (20) and IgA blistering diseases (21). The potential role of IgA autoantibodies in pathogenesis is, however, mostly ignored, which may be due to the lack of suitable mouse models. Since mice lack expression of an FcαRI homologue, *in vivo* studies investigating the role of FcαRI/IgA interactions in inflammation have been restricted, which also hampered investigating the *in vivo* role of FcαRI in IgA-mediated diseases. Evidence for a pathogenic role of FcαRI/IgA interactions is therefore limited.

Previously, it was demonstrated that transgenic mice, in which human FcαRI was expressed on monocytes/macrophages under the CD11b promotor, develop IgA nephropathy with macrophage infiltration in damaged glomeruli, due to deposits of soluble FcαRI/IgA complexes (16). In accordance, soluble FcαRI-IgA complexes were found in the serum and deposits in the kidneys of patients with IgA nephropathy (17). IgA autoantibodies may also play a pathogenic role in celiac disease, although this is still ill-understood. Patients with celiac disease can develop anti-tissue transglutaminase (tTG) IgA autoantibodies in response to gluten exposure (13). Mostly mononuclear cell infiltrates are found in the gastrointestinal tract of patients with celiac disease. The skin manifestation of celiac disease, referred to as dermatitis herpetiformis (DH), is characterized by the presence of IgA autoantibodies against tTG that are cross-reactive with epidermal TG, resulting in granular IgA deposits at the dermal-epidermal junction. Interestingly, this leads to the recruitment and activation of neutrophils rather than mononuclear cells (14).

Neutrophils are the most abundant circulating leukocytes in humans and play a fundamental role in innate immune responses. *In vitro* crosslinking of FcαRI on neutrophils induces pro-inflammatory responses, including the production of reactive oxygen species (ROS), release of cytokines, phagocytosis and the release of neutrophil extracellular traps (NETs) (22, 23). FcαRI crosslinking by IgA immune complexes also initiates the release of the neutrophil chemoattractant leukotriene B4 (LTB4) and concomitant neutrophil recruitment *in vitro* (24). Furthermore, serum of patients with the autoimmune blistering disease Linear IgA Bullous Disease (LABD) induced neutrophil-mediated tissue damage *in vitro* (25). As such, we hypothesize that the presence of IgA autoantibodies may result in neutrophil recruitment, but apart from these *in vitro* data, there are no studies examining the contributions of IgA autoantibodies and neutrophils to pathology in autoimmune diseases.

To investigate the *in vivo* role of neutrophil FcαRI in IgA-mediated disease, we developed a novel IgA autoimmune blistering model, which closely resembles human LABD by using genetically modified mice that produce human IgA and express human FcαRI. Our results provide the first evidence for a critical role of neutrophil FcαRI and IgA autoantibody interactions in activating neutrophils, resulting in accumulation of cells and pathogenic tissue damage *in vivo*. Blocking FcαRI effectively prevented neutrophil activation and decreased already existing inflammation, emphasizing the therapeutic potential of anti-FcαRI monoclonal antibodies in IgA-mediated diseases.

## Material and Methods

### Study Design

Human IgA anti-collagen XVII (mCOL17) antibodies were generated by immunizing human IgA knock-in mice (26) and (functionally) characterized. Anti-mCOL17 hIgA were injected during different time frames in ears of FcαRI transgenic mice or wild type littermates. The influx of neutrophils and the development of tissue damage and blisters was determined with several readouts in mice. In an alternative set of experiments mice were treated with anti-FcαRI or isotype controls mAbs.

### Generation of Hybridoma Cell Line Producing Anti-mCOL17 hIgA Antibodies

Three recombinant GST fusion fragments of the extracellular domain (GST-mCXVII-EC1, GST-mCXVII-EC3, GST-mCXVII-EC7) and one of the intracellular domain (GST-mCXVII-IC2) of mCOL17 were cloned and purified as previously published (27). hIgA knock-in mice (26) were immunized subcutaneously with 40 μg of a mixture of these four different constructs of mCOL17 in Complete Freund’s Adjuvant (Sigma-Aldrich. St. Louis, MO), followed by four booster injections of 40 μg of the mixture of recombinant mCOL17 in Incomplete Freund’s Adjuvant (Sigma-Aldrich). Mice were sacrificed at day 83 and mouse spleens were harvested. SP2/0 mouse myeloma cells were fused with splenocytes from immunized mice according to standard protocols (28). Positive hybridoma clones that produced anti-mCOL17 hIgA were selected by a mCOL17 hIgA ELISA (see below). To obtain monoclonal cell lines, cells from positive wells were subcloned by limiting dilution. Subcloning of monoclonal lines was repeated twice to make sure generated hybridoma lines were derived from single clones. Hybridomas were frozen in liquid nitrogen till further use. Anti-mCOL17 hIgA mAb were isolated using a S*taphylococcus aureus* superantigen-like protein 7 agarose (SSL7/Agarose) affinity column according to the manufacturers’ instructions (*In vivo*Gen, San Diego, CA).

### SDS-PAGE and Western Blot

Anti-mCOL17 hIgA and controls were prepared in reducing and non-reducing Laemmli sample buffer and analyzed by SDS-PAGE for detection of human IgA. Gels were stained with Silver Staining according to the manufacturers’ instructions (Thermo Scientific, Weltham, MA). Polyacrylamide gels were transferred on polyvinylidene fluoride (PVDF) membranes (Immobilon-P, Millipore corporation, Bedford, MA), and blocked with 5% BSA in Tris (hydroxymethyl)aminometane (TBS) for 1 hour at room temperature (RT). Membranes were probed with mouse anti-human IgA mAb (1:2000, BD Pharmingen™, Erembodegem, Belgium) for 1 hour at RT followed by incubation for 45 minutes with a secondary anti-mouse 800 Odyssey labelled antibody (1:150 000, Li-Cor Biosciences, Lincoln, NE). After extensive washing in TBST (TBS, 0.05% Tween-20), bound antibody was detected with an Odyssey Infrared Imaging System (Li-Cor Biosciences).

### Isolation of Human Neutrophils From Healthy Controls

Blood samples were commercially purchased at Bloodbank Sanquin (Amsterdam, The Netherlands). Neutrophils were isolated from human peripheral blood that was obtained from healthy donors using Lymphoprep (Axis-Shield, Oslo, Norway) density gradient centrifugation, after which erythrocytes were lysed in ammonium chloride buffer (155 mM NH_4_Cl, 10 mM KHCO_3_ and 0.11 mM EDTA, 10 minutes, RT). After lysis, neutrophils were washed with phosphate-buffered saline (PBS; B.Braun, Melsungen, Germany). Cells were resuspended in RPMI 1640 (Gibco BRL, Paisley, UK) that was supplemented with 10% FCS, glutamine and antibiotics. Neutrophils were labeled with calcein-acetoxymethylester (1 μmol/L; Molecular Probes Inc, Eugene, OR) for binding assays according to the manufacturer’s instructions.

### Ligand Binding Assay

Flat well microtitre ELISA plates (Nunc-Immuno MaxiSorp, Roskilde, Denmark) were coated with 100 µl anti-mCOL17 hIgA (10 µg/ml) or bovine serum albumin (10 µg/ml; BSA, negative control). After washing, wells were incubated with calcein labeled neutrophils (2.5 x 10^5^ cells/well) for 20 minutes at 37°C. Subsequently, supernatant was harvested and used for lactoferrin (degranulation marker for neutrophils) ELISA to investigate activation of neutrophils (see below). Plates were then washed and bound cells were lysed and fluorescence of supernatant (reflecting number of calcein-labeled neutrophils) was measured using a fluorimeter. A standard curve generated from 0-600.000 of calcein-labeled neutrophils was used to quantify neutrophil binding (485 nm excitation/520 nm emission filters; Fluostar Galaxy, BMG Labtechnologies, Offenburg, Germany).

### ELISA

#### Mouse Collagen Type XVII ELISA

Flat well microtitre ELISA plates (Nunc-Immuno MaxiSorp) were coated with a mixture of mouse collagen XVII EC1, EC3, EC7 and IC2 constructs (500 ng/well), followed by a blocking step for non-specific binding sites with PBS with Tween-20 (PBST, 200 µl) containing 0.5% BSA for 1 hour at 37 °C. Coated plates were incubated with diluted sera from mice or purified anti-mCOL17 hIgA (1 hour, 37 °C). Plates were washed with PBST, followed by incubation with biotin-labelled mouse anti-human IgA mAbs (1:250, 1 hour, 37° C, BD Biosciences, FranklinLakes, NJ). Plates were washed and further incubated with streptavidin horseradish peroxidase (HRP) (1:5000, 30 minutes, RT). As substrate (3,3’, 5,5’)-tetramethylbenzidine (TMB) was used. Plates were read with a microplate reader (Bio-Rad) at 450 nm.

#### Lactoferrin ELISA

Lactoferrin was measured in the supernatants of ligand binding assay. Flat well microtitre ELISA plates (Nunc-Immuno MaxiSorp) were coated with 100 µl rabbit anti-human lactoferrin mAb (50 µg/ml, Sigma) followed by a blocking step for non-specific binding sites with PBST containing 0.5% BSA for 1 hour at 37 °C. Plates were then incubated with 2x diluted supernatant for 1 hour at 37 °C, followed by incubation with alkaline phosphatase–labeled rabbit anti-human lactoferrin antibodies (1:2500; MP Biomedicals, LLC, Solon, OH) for 1 hour at 37 °C. Plates were washed between incubation steps with PBST. After addition of the chromogenic substrate P-nitrophenyl phosphate (Sigma), plates were read with a microplate reader (Bio-Rad) at 405 nm. Purified human lactoferrin (Sigma) was used as a standard to calculate the amount of lactoferrin in the measured samples.

#### Phagocytosis Assay

Carboxylate-modified polystyrene, green fluorescent latex beads (ø1.0 μM, Sigma-Aldrich) were washed twice with 2-(N-morpholino) ethanesulfonic (MES) buffer (30 mM, pH 6.1) and resuspended in MES buffer with 2 mg/ml BSA (Roche Diagnostics) or serum IgA (MP Biomedicals cat:0855906) in the presence of N-(3-Dimethylaminopropyl)-N’-acid ethylcarbodimide hydrochlorid (Sigma-Aldrich) and incubated o/n (overhead shaker, 4°C). Latex beads were washed and resuspended in PBS containing 0.1% BSA. BSA- or IgA- coated green fluorescent latex beads were incubated with neutrophils for 30 min at 37°C in effector:target (ET) ratios of 1:60. When indicated, neutrophils were pre-incubated with 60 mM molar whole or F(ab’)_2_ anti-FcαRI (MIP8a, BioRad, cat:MCA1824EL) antibodies for 30 min at 4°C. MIP8a F(ab’)_2_ antibodies were produces from whole MIP8a according to the manufacturer’s instructions (Thermoscientific, cat:RB227015A). After washing, fluorescence was measured with flow cytometry (LSR-Fortessa X20, BD Bioscience). The phagocytic index was calculated by multiplying the percentage of cells that had taken up beads with mean fluorescence intensity of bead-positive cells. Data was analyzed with Flowjo software (Tree Star).

#### Calcium-Flux Assay

Peripheral neutrophils were loaded with calcium indicator Fluo-4-AM (3 μM, Invitrogen, cat:F14207) in complete medium at 37°C for 30 min. After washing with medium, cells were allowed to settle for 30 min at 37°C. Neutrophils were incubated with 4 mg/ml monomeric anti-mCOL17 hIgA antibodies for 30 min on ice. Cells were washed and 3x10^5^ cells were seeded per condition in a 96-well plate (FLUOTRAC 200; Greiner Bio-One). Secondary goat α human IgA antibodies (Jackson Immuno Search, cat:109-006-011) were added directly into 96-well plates. Immediately, fluorescent signal was measured every 10 seconds for 20 min in a preheated fluorometer at 37°C at 485 nm excitation, 520 nm emission (FLUOstar/POLARstar; BMG Labtech).

#### Western Blotting

To prepare crude cell lysates, 2*10^6^ neutrophils per condition were washed and resuspended in RPMI without serum. Subsequently, cells were incubated with 10 mg/ml anti-mCOL17 hIgA antibodies for 30 min on ice. Cells were crosslinked using 300 µg goat α human IgA (Jackson Immuno Search, cat:109-006-011) at 37°C for a time course of 0, 1, 2, 3, 5, 10 and 20 min. Immediately after the indicated time points, cells were boiled for 10 min at 95°C in pre-heated 4X Laemmli buffer containing 8% 2-mercaptoethanol. Samples were thoroughly vortexed, spun down and kept at -20°C until usage. For western blotting, samples were loaded on a 10% polyacrylamide gel according to standard procedures. Transfer occurred using the semi-dry iBlot system (Novex, Life Technologies, Paisley, U.K.). Membranes were blocked in 5% BSA in PBS-Tween 0.05% for 1 hour. Membranes were incubated O/N with 1:3000 GAPD (14C10, Cell Signaling, cat: 21185), 1:1000 total ERK (Cell Signalling, cat:9102), 1:1000 phospho-ERK (Cell Signalling, 4377S). Membranes were developed using an ECL reagent (ThermoFischer) with a Chemidoc Imaging Touch system (Bio-Rad). Quantification of phosphorylation was determined with ImageJ (U. S. National Institutes of Health, Bethesda, MD).

### Mice

Transgenic mice that express human FcαRI on neutrophils (29) were backcrossed onto BALB/c background. Mice were further crossed with either mice expressing enhanced green fluorescent protein (LysEGFP) on neutrophils (30) for intravital imaging experiments, or with mice that were hIgA knock-in (26). Obtained LysEGFP, FcαRI/LysEGFP, hIgA, and FcαRI/hIgA BALB/c mice were bred and housed at the Central Animal Facility of the VU University (Amsterdam, The Netherlands) under standard conditions. All ear injections were performed after mice were narcotized by administration of a mixture of Ketamine/Xylazine anesthesia. Experiments were performed according to institutional and national guidelines. The animal ethical committee of the VU University Medical Center approved all experiments.

#### Intravital Imaging

Intravital imaging of blood vessels was used to examine the potential of anti-mCOL17 hIgA to activate neutrophils *in vivo*. Mouse ears are optimal imaging sites as they are thin and relatively transparent allowing non-invasive imaging of the blood circulation to analyze neutrophil activation and migration. For intravital imaging, anti-mCOL17 hIgA mAb (35 µg; right ear) or PBS (left ear) was injected intracutaneously in ears of LysEGFP or FcαRI/LysEGFP transgenic mice. After 48 hours, mice were narcotized by administration of a mixture of Hypnorm/Dormicum and intravital imaging recordings were performed with a ZEISS Axiovert 200 Marianas inverted microscope (Marianas, I.I.I., Denver, CO). A cooled EM-CCD camera (Photometrics, Tucson, AZ; 512 x 512 pixel) recorded images with 16-bit capability. Imaging was performed with a 10X air lens (ZEISS) and standard GFP filter set. In total 2400 frames were taken with an interval of 0.5 second (20 minutes). After the recordings, injected ears were harvested and snap frozen for immunofluorescence staining.

#### *In vivo* Inflammation Experiments

For *in vivo* inflammation experiments, FcαRI/hIgA or control hIgA mice received anti-mCOL17 hIgA (70 µg) subcutaneously every other day for 1 week (4 injections in total) or for 14 days (7 injections in total) (**Supplementary Figure 2** for injection scheme). Additionally, in some experiments mice were treated intraperitoneally with 100 μg anti-FcαRI mAb (MIP8a; Serotec, Uden, the Netherlands) on day 0 or day 3 (prevention model) with 150 µg on day 7 or day 11 (treatment model). Control mice received an isotype control (Ultra-LEAF™ Purified Mouse IgG1, κ Isotype Ctrl Antibody, Biolegend) at the same days. Mice were monitored daily for discomfort and at the end of the experiments, ears were harvested and snap frozen to use for immunofluorescence staining.

### Immunofluorescence

Mouse ear cryosections (6-8 μm) of FcαRI/hIgA transgenic mice were fixed in acetone and air-dried, after which they were incubated with purified anti-mCOL17 hIgA (1 hour, RT), followed by washing and incubation with anti-hIgA FITC mAb (1:40, DAKO, Heverlee, Belgium). Alternatively, ear cryosections of LysEGFP, FcαRI/LysEGFP, hIgA and FcαRI/hIgA transgenic mice were stained with anti-mouse GR-1 FITC mAb (1:100, eBioscience, San Diego, CA; 1 hour, RT) or with anti-hIgA FITC mAb (1:40, DAKO; 1 hour, RT). Diamidino-2-phenylindole (DAPI) was used for nuclear staining. Cryosections were analyzed with a Leica DM6000 microscope (Leica Microsystems B.V.). Tile scanning scan was performed to obtain a composite image of the whole ear. Additional analyses were performed using ImageJ software measuring ear thickness and RawIntDen/Area of GR-1 staining.

### Quantification of Images

GR-1 staining of cryosections was analyzed using ImageJ. Tile scans of whole ears were taken for analysis. Region of interests were determined based on DAPI staining. The raw integral density (RawIntDen, the sum of pixels in the image) was measured for the GR-1 staining. Quantification was calculated as the RawIntDen of GR-1 staining divided by the area of the DAPI staining.

### Online Supplemental Material

**Figure 1:**
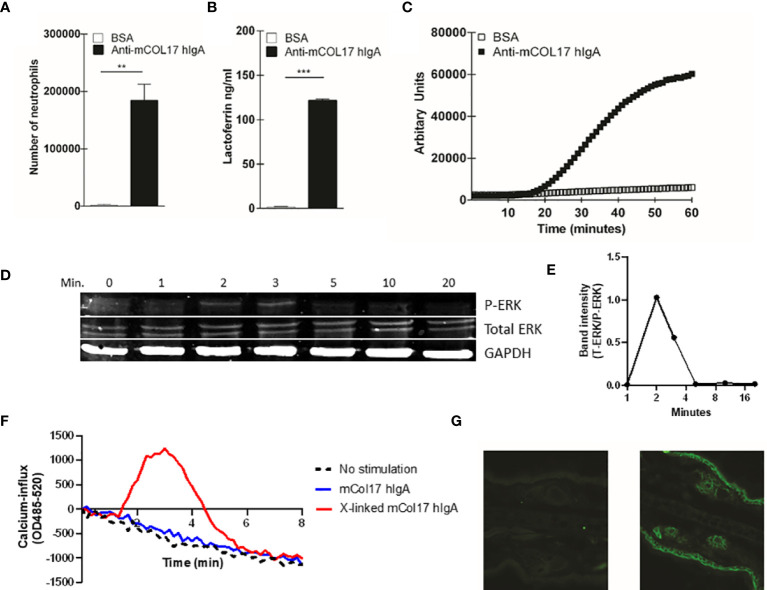
Anti-mCOL17 hlgA antibodies activate neutrophils *in vitro*. **(A)** Binding of fluorescently-labeled neutrophils to BAS or anti-mCOL17 hlgA- coated wells. **(B, C)** Secretion of **(B)** lactoferrin (as measure of degranulation) or **(C)** ROS production after addition of neutrophils to wells coated with BSA or anti-mCOL17 hlgA. **(D, E)** FcαRI on peripheral human neutrophils was stimulated with anti-mCOL17 hlgA antibodies and cross-linked for 0, 1, 2, 3, 5, 10 and 20 minutes. **(D)** Western blotting of phosphorylated ERK (upper panel), total ERK (middle panel) and household protein GAPDH (lower panel). Results from one donor are shown, being representative image of three donors. **(E)** Quantification of the ratio total ERK protein divided by phosphorylated ERK protein at indicated time points of a single donor. **(F)** Human neutrophils were incubated with anti-mCOL17 hlgA antibodies. Release of intracellular calcium over time was measured with (red line) or without (blue line) cross-linking. Unstimulated neutrophils served as negative control (black dotted line). **(G)** Human IgA staining (green fluorescence) of mouse ear cryosections incubated with (non-specific) pooled human serum IgA (left panel) or anti-mCOL17 hlgA (right panel). Scales bars: 50 μm. Data are presented as mean ± SD. Student’s *t* test; **p < 0.001, ***p < 0.0001.

Supplemental material contains the movies of intravital imaging. In all videos, blood vessels of mouse ears were recorded. LysEGFP neutrophils are displayed in green. Videos of blood vessels were taken 48 hours after injection of anti-mCOL17 hIgA antibodies or PBS in the ears of mice. **Figure 1** and **Supplementary Video 1** demonstrates ears of LysEGFP mice after injection with PBS or anti-mCOL17 hIgA. **Figure 1** and **Supplementary Video 2** demonstrates ears of FcαRI/LysEGFP mice after injection with PBS, anti-mCOL17 hIgA or with anti-mCOL17 hIgA in combination with anti-FcαRI monoclonal antibody therapy.

### Statistical Analysis

Data analysis was performed using GraphPad Prism version 4.03 for Windows (GraphPad Software, San Diego, CA). Data are expressed as mean ± SD. Statistical differences were determined using two–tailed unpaired Student’s *t* tests (comparing 2 groups) or ANOVA (> 2 groups). Differences were considered statistically significant if p < 0.05.

### Study Approval

The Medical Ethical Committee (METC) of VU University Medical Center (The Netherlands) reviewed the study, agreed with the blood sample collection and approved the informed consent form. All donors gave written informed consent, in accordance with the guidelines of the METC and the Declaration of Helsinki. The animal ethical committee of the VU University Medical Center approved all mice experiments, and experiments were performed according to institutional and national guidelines.

## Results

### Generation of Specific Human IgA Anti-Mouse Collagen XVII Antibodies

In order to investigate the *in vivo* role of neutrophil FcαRI in IgA-mediated disease, we made use of a transgenic mouse model in which human FcαRI is expressed on neutrophils (29), as mice do not express FcαRI. Human FcαRI only binds efficiently to human IgA (hIgA), but poorly interacts with murine IgA. We therefore generated hIgA anti-mouse collagen XVII [mCOL17; the target auto-antigen in LABD (31)] by immunizing hIgA knock-in mice (26) with proteins of the extracellular and intracellular domain of mouse collagen XVII (27). After successful immunization, anti-mCOL17 hIgA hybridoma cell lines were generated and the antibodies were characterized by ELISA and western blot (**Supplementary Figures 1A–C**).

To investigate whether anti-mCOL17 hIgA antibodies were functional, we first performed a ligand binding assay to examine neutrophil binding, degranulation and ROS production. FcαRI-expressing neutrophils show firm binding to anti-mCOL17 hIgA antibodies (**Figure 1A**). Furthermore, neutrophils released lactoferrin, which is indicative of degranulation (**Figure 1B**) and produced ROS after binding to anti-mCOL17 hIgA antibodies (**Figure 1C**). Additionally, we investigated whether cross-linking of anti-mCOL17 hIgA mediated FcαRI-induced ITAM activated signaling, by measuring phosphorylation of extracellular signal-regulated kinase (ERK) and the induction of release of intracellular calcium (32, 33). FcαRI cross-linking by anti-mCOL17 hIgA induced phosphorylation of ERK, which was observed after 2 and 3 minutes (**Figure 1D, E**). Furthermore, FcαRI cross-linking by anti-mCOL17 hIgA induced release of intracellular calcium in neutrophils (**Figure 1F**), supporting that mCOL17 hIgA antibodies stimulated the ITAM activating pathway. To investigate whether anti-mCOL17 hIgA antibodies recognize mouse collagen XVII *in situ*, we incubated mouse ear dermis with the newly generated antibodies. Immunofluorescence (IF) microscopy confirmed binding of anti-mCOL17 hIgA to keratinocytes and hair follicles (**Figure 1G**). Anti-mCOL17 hIgA antibodies did not cross-react with epitopes in human skin (data not shown). Taken together, these results showed that the obtained anti-mCOL17 hIgA antibodies were specific for mouse collagen XVII and able to activate FcαRI-expressing neutrophils *in vitro*.

### Anti-mCOL17 hIgA Induces Neutrophil Migration in Mice Expressing Human FcαRI

Neutrophils need to extravasate from the circulation into the tissues to exert their antimicrobial function or to induce tissue damage in case of disease (34). Therefore, we first used intravital imaging of blood vessels to examine the potential of anti-mCOL17 hIgA to activate neutrophils *in vivo*. Mouse ears are optimal imaging sites as they are thin, relatively transparent and no surgical procedures are required for imaging. This allows non-invasive intravital imaging of the blood circulation to analyze neutrophil activation and migration.

**Figure 2:**
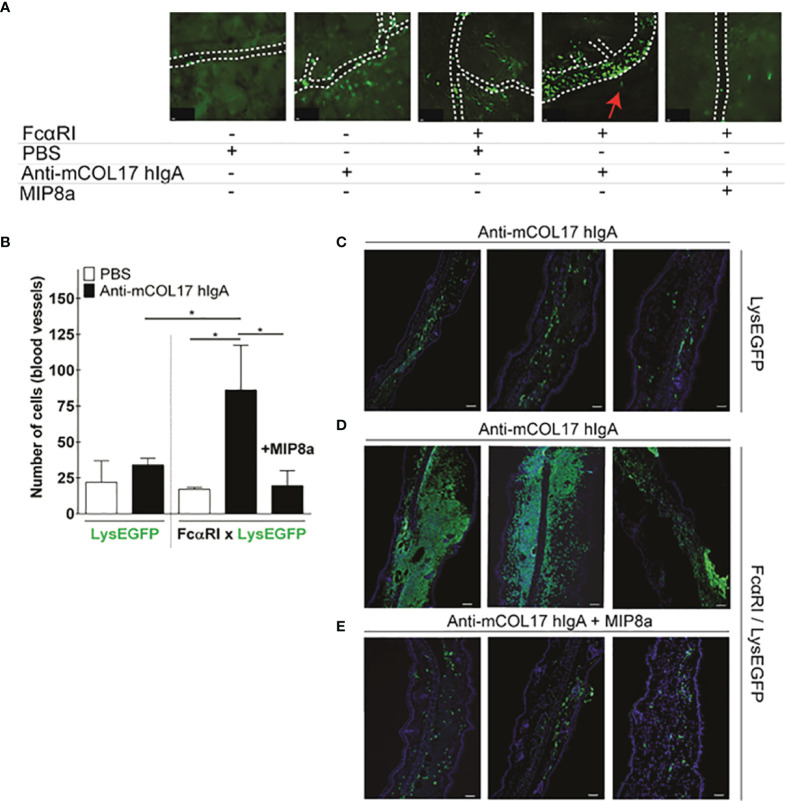
Anti-mCOL17 hlgA antibodies induce FcαRI-dependent neutrophil recruitment. **(A)** Still frames from intravital imaging movies, 48 hours after injection of PBS (first panel) or anti-mCOL17 hlgA (second panel) in ears of LysEGFP mice. Ears of FcαRI/LysEGFP mice were injected with PBS (third panel), anti-mCOL17 hlgA (fourth panel) or anti-mCOL17 hlgA in combination with systematic treatment with FcαRI blocking mAb MIP8a (fifth panel). Red arrowheah indicates neutrophil extravasation (see also **Supplementary Videos 1** and **2**). Scale bars: 10 μm. **(B)** Quantification of neutrophil numbers in contact with blood vessels in intravital imaging movies. n = 3 per group. **(C–E)** Cryosection of ears of **(C)** LysEGFP mice injected with anti-mCOL17 hlgA, **(D)** FcαRI/LysEGFP mice injected with anti-mCOL17 hlgA without or **(E)** with treatment with anti-FcαRI mAb MIP8a. Three examples per group are shown. Cryosections were stained with the neutrophil marker GR-1 (green) and DAPI (DNA; blue). Scale bars: 75 μm. Data are presented as mean ± SD. ANOVA; *p < 0.05.

To monitor neutrophil activation *in vivo*, we used LysEGFP mice, in which endogenous neutrophils are bright fluorescent green (30). LysEGFP mice, lacking the expression of FcαRI on neutrophils, were injected with anti-mCOL17 hIgA antibodies in the right ear and with PBS in the left ear. Intravital imaging of ears was performed after 48 hours. Minimal neutrophil activation and recruitment was observed in ears of LysEGFP mice that had been injected with either PBS or anti-mCOL17 IgA (**Figures 2A, B** and **Supplementary Video 1**). Similarly, minimal neutrophil activation was observed in ears of FcαRI/LysEGFP mice that had been injected with PBS (**Figures 2A, B** and **Supplementary Video 2**). By contrast, when FcαRI/LysEGFP mice were injected with anti-mCOL17 hIgA antibodies, neutrophils accumulated at the blood vessel wall, and migration and extravasation into ear tissue was observed (**Figures 2A, B** and **Supplementary Video 2**).

IgA-induced neutrophil activation and migration was completely prevented in mice that received a FcαRI blocking antibody (MIP8a) (**Figures 2A, B** and **Supplementary Video 2**). The severity of inflammation was scored by quantifying the number of recruited neutrophils into the ear dermis after injection of anti-mCOL17 hIgA. When cryosections of the injected ears were analyzed *ex vivo*, some neutrophil influx was observed in the ears of control LysEGFP mice (**Figure 2C**). However, FcαRI/LysEGFP mice that were injected with anti-mCOL17 hIgA showed inflammation of the ears characterized by massive accumulation of neutrophils into the ear dermis (**Figure 2D**). By contrast, FcαRI/LysEGFP mice that had additionally been treated with the FcαRI blocking antibody showed minimal neutrophil influx in the ears (**Figure 2E**) and cannot be distinguished from the controls (**Figure 2C**). Theoretically, inhibition of migration may have been due to engagement of the Fc tail of MIP8a with the inhibitory receptor FcγRII. However, when neutrophils were incubated with IgA-coated particles, phagocytosis was inhibited by both MIP8a and MIP8a F(ab’)_2_ (**Supplementary Figure 3A**), indicating that blocking phagocytosis of IgA-complexes is independent of the Fc portion of MIP8α. This supports that the inhibitory FcγRIIb does not contribute to alleviation of mCOL17 hIgA-mediated pathology after mAb therapy, but indicates that inhibition of migration is mediated by blocking the interaction between IgA and FcαRI.

### IgA Autoantibody-Induced Neutrophil Accumulation Leads to Tissue Damage

**Figure 3:**
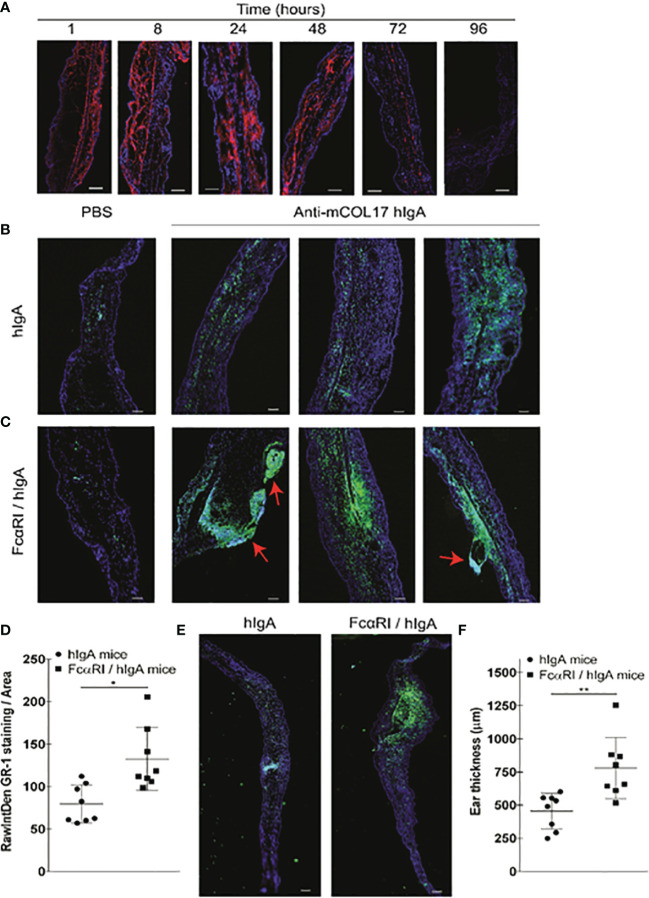
hlgA anti-mCOL17-induced neutrophil migration and chronic inflammation in FcαRI/hlgA mice. **(A)** Cryosections of ears of FcαRI/hlgA mice were stained with anti-human lgA (red) and DAPl (DNA; blue) after injection of anti-mCOL17 hlgA antibodies. Mice were sacrificed at indicated time points. Scale bars: 75 μm. **(B, C)** hlgA **(B)** and FcRI/hlgA **(C)** mice were injected with PBS (left panel) or anti-mCOL17 hlgA (right panels) every other day till sacrifice on day 7 (in total 4 injections). Cryosections were stained with the neutrophil marker GR-1 (green) and DAPl (DNA; blue). 3 representative examples are shown. Red arrowheads indicate blister formation and tissue damage. Scale bars: 100 μm. **(D)** Quantification of GR-1 staining. Each dot represents one mouse. N = 8 per group **(E, F)** Analysis of ear thickness. **(E)** A representative cryosection example of a hlgA mouse ear (left panel) or FcαRI/hlgA mouse ear (right panel) are shown. Scale bars: 250 μm. **(F)** Quantification of ear thickness. N = 8; each dot represents one mouse. Data are presented as mean ± SD. Student’s *t* test; *p < 0.05, **p < 0.001.

Long-term exposure to IgA-antigen complexes is required to initiate cell activation and ultimately to induce chronic inflammation. Unfortunately, hIgA has a short half-life in mice (35). As such, we first determined the stability of our IgA antibodies *in vivo* by defining the presence of antibody within the ear dermis over time after a single dose injection of anti-mCOL17 hIgA antibodies. hIgA was observed up to 48 hours after injection, after which it was rapidly removed and no longer present after 72 and 96 hours (**Figure 3A**). To mimic chronic inflammation, we injected the right ears of FcαRI/hIgA or hIgA mice with anti-mCOL17 hIgA and left ears with PBS 4 times every other day. Mice were sacrificed at day 7, and we investigated whether IgA-induced neutrophil activation and migration induced tissue damage.

Low numbers of neutrophils were seen in ears injected with PBS in either hIgA or FcαRI/IgA mice as demonstrated by microscopic analysis of ear cryosections (**Figures 3B, C**, left panels). Frequent injection of anti-mCOL17 hIgA in ears of hIgA mice induced some neutrophil influx (**Figure 3B**, right panels). However, massive recruitment and accumulation of neutrophils was observed after frequent injection of anti-mCOL17 hIgA in ears of FcαRI/hIgA mice (**Figure 3C**, right panels and 3 D). Ear thickness was measured to assess the degree of inflammation after injection of anti-mCOL17 hIgA antibodies. Ears of FcαRI/hIgA mice that had been injected with anti-mCOL17 hIgA showed increased thickness when compared to ears of hIgA mice (**Figures 3E, F**). Importantly, in addition to augmented accumulation of neutrophils, also tissue damage and blisters were observed in ears of FcαRI/hIgA mice in response to multiple injections of anti-mCOL17 hIgA (**Figure 3C**). Thus, continuous presence of IgA-antigen complexes in ears of FcαRI transgenic mice induced chronic inflammation and tissue damage mediated by neutrophils *in vivo*.

### Blocking Anti-FcαRI Monoclonal Antibodies Prevent IgA Autoantibody-Induced Neutrophil Accumulation

**Figure 4:**
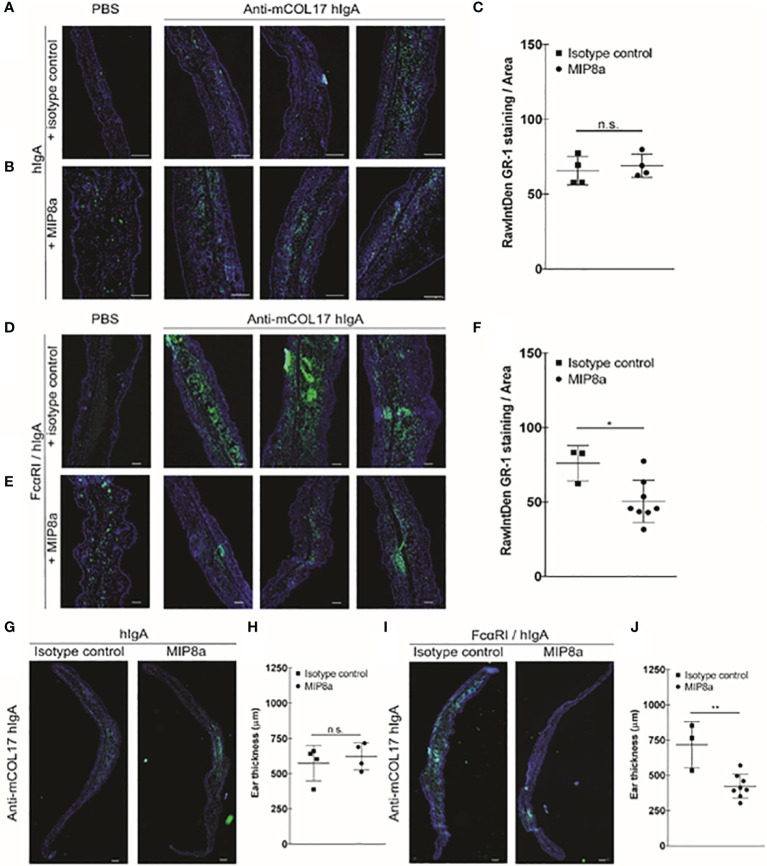
Blocking FcαRI prevents lgA-induced neutrophil accumulation and thickening ears. **(A, B)** hlgA and **(D, E)** FcαRI/hlgA mice were injected with PBS (left panels) or anti-mCOL17 hlgA (right panels, 3 examples) every other day till sacrifice on day 7 (in total 4 injections) in combination with a **(A, D)** isotype control antibody or **(B, E)** anti-FcαRI mAb MIP8a **(C, F)** Quantification of GR-1 staining. **(G, I)** Analysis of ear thickness of **(G)** hlgA or **(I)** FcαRI/hlgA mice after treatment with an isotype control (left panel) or MIP8a (right panel). A representative example of a mouse ear cryosection is shown. **(H, J)** Quatification of ear thickness. Each dot represents one mouse. hlgA mice (isotype control or MIP8a) n = 4; FcαRI/hlgA mice (isotype control) n = 3; FcαRI/hlgA mice (MIP8a) n = 8. Cryosection of ears were stained with the neutrophil marker GR-1 (green) and DAPl (DNA; blue). Scale bars: 250 μm **(A, B, G, I)** or 100 μm **(D, E)**. Data are presented as mean ± SD. Student’s *t* test; *p < 0.05, **p < 0.001. n.s., not significant.

Since frequent injection of anti-mCOL17 hIgA induced neutrophil activation in FcαRI/hIgA mice, we next addressed the question whether blocking the interaction between IgA and FcαRI prevents accumulation of neutrophils and concomitant chronic inflammation. Next, to address whether blocking the interaction between IgA and FcαRI prevents accumulation of neutrophils and concomitant chronic inflammation *in vivo*, we injected mice with anti-mCOL17 hIgA or PBS every two days. To block FcαRI on neutrophils, mice were injected intraperitoneally with an FcαRI blocking monoclonal antibody (mAb). Control mice received an isotype control mAb. Mice were sacrificed after 4 injections on day 7. No differences in neutrophil recruitment were found after co-injection with an isotype control (**Figures 4A, C**) or anti-FcαRI mAb MIP8a (**Figures 4B, C**) in hIgA mice. Low numbers of neutrophils were present in ears of FcαRI/hIgA mice that had been injected with PBS (**Figures 4D, E**, left panels). However, massive neutrophil accumulation was observed in earsof FcαRI/hIgA mice that had been injected with anti-mCOL17 hIgA, and received an isotype control antibody (**Figure 4D**, right panels). Importantly, blocking FcαRI reduced neutrophil recruitment and accumulation to control levels (**Figure 4E**, right panels). Quantification of tile scans of cryosections of ears demonstrated significantly less neutrophil accumulation after treatment with an FcαRI blocking antibody when compared to isotype control group (**Figure 4F**). Additionally, injection of MIP8a in hIgA mice had no effect on ear thickness, as compared with injection of an isotype control (**Figures 4G, H**). However, treatment with an FcαRI blocking mAb resulted in a significant decrease in ear thickness in FcαRI/hIgA mice after injection of anti-mCOL17 hIgA compared to mice that received an isotype antibody (**Figures 4I, J**).

### Anti-FcαRI Monoclonal Antibodies as Novel Therapeutic Target for IgA-Induced Chronic Inflammation

**Figure 5:**
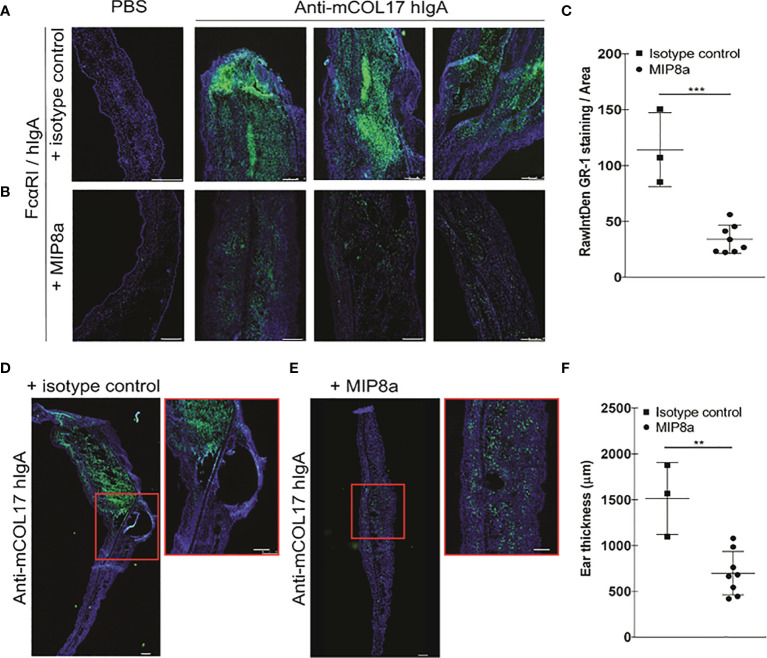
Anti-FcαRI mAbs significantly reduces lgA-induced neutrophil recruitment, existing chronic inflammation and tissue damage. FcαRI/hlgA mice were injected with PBS (left ear) or anti-mCOL17 hlgA (right ear) every other day till sacrifice on day 14 (in total 7 injections). Mice were treated with an isotype antibody or anti-FcαRI mAb MIP8a at day 8 and 11. **(A, B)** FcαRI/hlgA mice were treated with an isotype control **(A)** or with MIP8a **(B)**. Left panels; PBS injection. Three representative examples of ears that had been injected with anti-mCOL17 hlgA are shown. **(C)** Quatification of GR-1 staining. **(D-F)** Analysis of ear thickness. A representative example of mouse ear cryosection is shown after treatment with an isotype control **(D)** or MIP8a **(E)**. Inserts are magnifications. **(F)** Quantification of ear thickness. Each dot represents one mouse. Isotype control n = 3; MIP8a n = 8. Cryosections of ears stained for the neutrophil marker GR-1 (green) and DAPI (DNA; blue). Scale bars: 250 μm. Data are presented as mean ± SD. Student’s *t* test; **p < 0.05, ***p < 0.001.

Thus, blocking FcαRI prevented IgA autoantibody-induced chronic inflammation in FcαRI/hIgA mice. However, patients with IgA-mediated autoimmune diseases only benefit from a therapy that decreases existing active disease. We therefore next investigated whether anti-FcαRI antibodies were able to diminish pre-existing IgA-induced chronic inflammation. FcαRI/hIgA mice first received 4 injections of anti-mCOL17 hIgA antibodies in the ears, which induced neutrophil accumulation and concomitant chronic inflammation at day 7 (for reference see **Figure 3**). At day 8 and 11, mice were treated with the FcαRI blocking antibody MIP8a or an isotype control. Injections with anti-mCOL17 hIgA or PBS continued every 2 days until the mice were sacrificed on day 14. Massive neutrophil accumulation was observed in ears of FcαRI/hIgA mice that had been injected with anti-mCOL17 hIgA, and had been treated with an isotype control antibody (**Figures 5A, C**). However, anti- FcαRI monoclonal antibody therapy resolved pre-existing inflammation after 7 days of treatment (**Figures 5B, C**). Additionally, prominent differences in ear thickness and tissue damage were observed between mice that had been treated with anti-FcαRI mAbs or mice which had received an isotype control. Mice treated with an isotype control antibody had had an ear thickness of on average 1500 µm, and significant tissue damage and blister formation was observed. However, treatment with an FcαRI antibody reduced ears thickness to ~ 600 µm (**Figures 5D–F**).

Taken together, anti-FcαRI blocking mAbs resolved pre-existing IgA-autoantibody-induced inflammation in mice, which emphasizes the therapeutic potential of anti-FcαRI antibodies for IgA-mediated diseases.

## Discussion

IgA autoantibodies or immune complexes and influx of neutrophils is observed in multiple diseases, including IgA vasculitis, ulcerative colitis, rheumatoid arthritis and skin blistering diseases (12, 24, 31, 36). We now provide *in vivo* evidence that demonstrates a causal relationship between the presence of IgA autoantibodies and FcαRI-induced neutrophil recruitment and activation, resulting in massive tissue damage. Importantly, treatment with an FcαRI blocking mAb prevented disease, but was also able to resolve existing inflammation, identifying anti-FcαRI mAb therapy as a novel strategy to counteract IgA-induced neutrophil accumulation and tissue damage.

Neutrophils are the first to be recruited to inflammatory sites and are capable of eliminating pathogens (36). Crosslinking of FcαRI triggers neutrophil activation, which leads to efficient phagocytosis of IgA-opsonized bacteria, release of pro-inflammatory mediators, and neutrophil recruitment through release of LTB4 (22, 24). This process likely plays an important role in mucosal defense to clear infections. However, activated neutrophils can also exacerbate inflammation and induce tissue damage. We hypothesize that neutrophil FcαRI might therefore play a detrimental role in IgA-mediated autoimmune diseases.

The autoimmune skin disorder LABD is associated with the presence of IgA autoantibodies directed against collagen XVII and neutrophil influx (37). We previously demonstrated that serum of LABD patients (containing IgA autoantibodies) induces neutrophil FcαRI mediated tissue damage *in vitro*, which supports our hypothesis although *in vitro* assays do not necessarily reflect the more complex pathology in autoimmune diseases (25). We now show a direct correlation between the presence of IgA autoantibody-induced neutrophil accumulation and the pathogenesis of blister formation *in vivo.* Importantly, treatment with anti-FcαRI blocking antibodies prevented mCOL17-induced LABD pathology. This was presumably due to direct interference with binding of IgA to FcαRI, and not due to inhibitory signaling *via* FcγRII.

Neutrophil-mediated chronic diseases are currently difficult to treat, which involves general suppression of immune responses with corticosteroids and immunosuppressive drugs or dapsone (38). In most cases, long-term treatment is necessary, making the side-effects of these therapies considerable and a major disadvantage as they are often poorly tolerated. Removing pathogenic autoantibodies by immunoadsorption or immunoapheresis is another strategy (39), but this is an intensive treatment because patient’s plasma needs to be filtered multiple times. A more specific treatment targeting the mechanism of action of IgA-mediated diseases may therefore be greatly beneficial for patients. For skin diseases it might be preferential to develop an ointment containing IgA or FcαRI peptide mimetics that block the interaction between IgA autoantibodies and neutrophil FcαRI (40). Peptides are small, and may penetrate the inflamed skin. Blocking anti-FcαRI mAbs will be more suitable for treatment of oral blisters that are commonly observed in LABD, or systemic manifestations of IgA-induced neutrophil recruitment in other diseases.

For instance, large FcαRI-positive neutrophils infiltrates are found in colons of patients with ulcerative colitis. Moreover, we previously observed that these neutrophils had taken up IgA complexes, supporting that IgA-mediated neutrophil migration may contribute to massive neutrophil recruitment in active disease (24). It has been reported that the intestinal microbiota is a key driver of inflammatory responses in IBD (41, 42). It was furthermore demonstrated that injection of highly IgA-coated colitogenic intestinal bacteria of IBD patients exacerbated the development of colitis in gnotobiotic mice (43). This study suggests that high IgA coating may identify commensals that drive intestinal disease in humans (44).

The role of IgA in other intestinal diseases is less clear. In patients with celiac disease, exposure to gluten-containing food results in the production of IgA autoantibodies. Yet, a mostly mononuclear cell infiltrate is observed in diseased small intestine, and neutrophils are not considered to play an important role in this disease (13). However, when celiac disease manifests in the skin, which is referred to as dermatitis herpetiformis, both IgA deposits and infiltration of neutrophils are found (45). It is ill-understood why IgA-induced neutrophil migration may be involved in skin, but not in gastrointestinal manifestations of the same disease, even though IgA autoantibodies against tTG are found at both locations.

Similarly, IgA nephropathy is characterized by accumulation of aberrant glycosylated IgA1 immune complexes in the glomerular mesangium (46). Shedding of FcαRI from monocyte cell membranes results in the soluble form of the receptor sFcαRI, and patients with IgA nephropathy have IgA-sFcαRI complexes in the circulation and deposits in the kidney (16). Monocytes and macrophages have been implicated in the pathogenesis of IgA nephropathy, but IgA-induced neutrophil activation does not seem to play a role, possibly because sFcαRI blocks the binding site for neutrophil FcαRI.

While IgA autoantibodies are obvious players in LABD, the involvement of IgA in autoimmunity might be broader than previously suspected. We previously demonstrated that IgA immune complexes that are present in plasma and synovial fluid of patients with rheumatoid arthritis activated neutrophils *in vitro*, resulting in production of ROS, release of NETs, lactoferrin and chemotactic stimuli (47). Neutrophils are found in high numbers in the synovial fluid of patients with rheumatoid arthritis (36), and we showed that auto-IgA complexes recruit and activate neutrophils *in vitro*. Moreover, we recently showed that IgA autoantibodies induced release of IL-6 and IL-8 by immune cells as well as osteoclasts, which enhanced bone resorption by osteoclasts (48). Thus, a role for IgA in inducing joint damage and exacerbation of disease is highly likely. This is supported by several studies, which showed a correlation between the presence of IgA rheumatoid factor or anti-citrullinated protein antibodies in blood or synovial fluid and worse disease progression including cartilage and bone erosion (19, 49–52).

**Figure 6:**
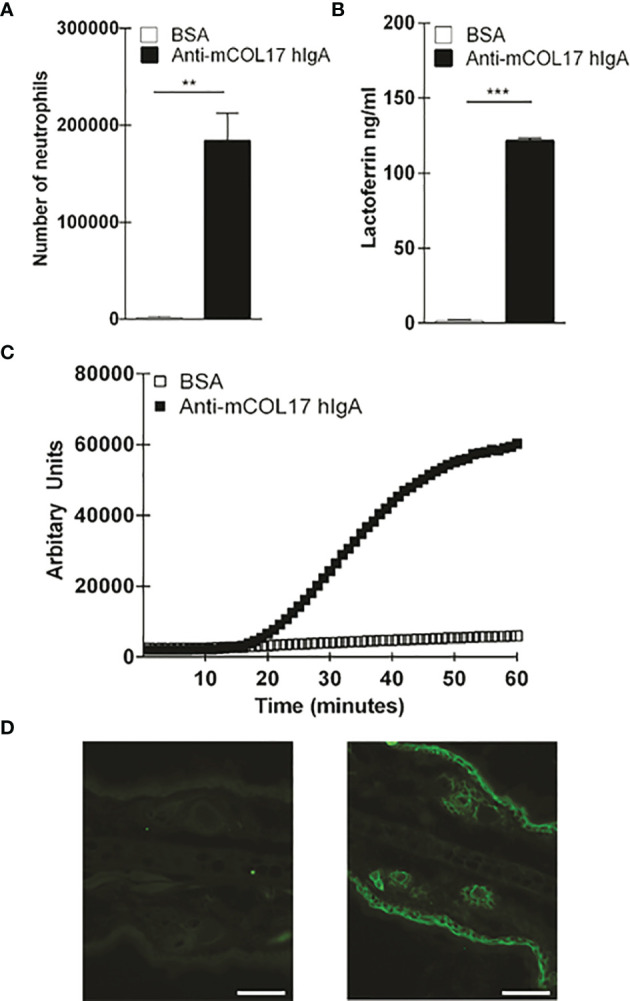
Anti-mCOL17 hIgA antibodies active neutrophils in vitro. **(A)** Binding of fluorescently-labeled neutrophils to BSA or anti-mCOL17 hIgA-coated wells. **(B, C)** Secretion of **(B)** lactoferrin (as measure of degranulation) or **(C)** ROS production after addition of neutrophils to wells coated with BSA or anti-mCOL17 hIgA. **(D)** Huma IgA staining (green flourescene) of mouse ear cryosection incubated with (non-specific) pooled human serum IgA (left panel) or anti-mCOL17 hIgA (right panel). Scale bars: 50um. Data are presented as mean ± SD. Student's t test; **p < 0.001, ***p < 0.0001.

Building on the reported data, we propose the following model of action in IgA-mediated autoimmune diseases or chronic inflammation (with LABD as example; **Figure 6**). IgA-antigen complexes in the tissue directly contribute to activation and recruitment of neutrophils through interaction with FcαRI. Newly recruited neutrophils will also encounter IgA-antigen complexes and become activated, which will lead to secretion of more chemoattractants, resulting in amplification of neutrophil migration. The accumulation of activated neutrophils will subsequently result in tissue damage (**Figure 6A**). Importantly, existing inflammation is resolved after treatment with anti-FcαRI mAbs (**Figure 6B**).

Collectively, our novel findings show that the consensus dogma of IgA as innocuous molecule needs to be revised, as IgA autoantibodies and neutrophil FcαRI interactions play a critical role in inducing neutrophil activation and accumulation, with concomitant severe tissue damage. Although it not yet understood why patients develop IgA autoantibodies, blocking FcαRI will help to dampen inflammation and limit tissue destruction in patients with IgA-mediated autoimmune diseases.

## Data Availability Statement

The raw data supporting the conclusions of this article will be made available by the authors, without undue reservation.

## Ethics Statement

The studies involving human participants were reviewed and approved by the Medical Ethical Committee of the VU University Medical Center. The patients/participants provided their written informed consent to participate in this study. The animal study was reviewed and approved by the animal ethical committee of the VU University Medical Center.

## Author Contributions

ME supervised the study. EA, LS, NH, SB, JB, ER, and SP performed experiments and analyzed data. PW contributed to rebuttal experiments. LB produced and purified IgA anti-mouse collagen XVII antibodies. RM provided expert help with intravital imaging. MC provided the hIgA knock-in mice. CS performed experiments and provided the constructs for mouse collagen XVII. ME, EA, and AB wrote the paper. All authors contributed to the article and approved the submitted version.

## Funding

This work was supported by the Netherlands Organization for Scientific Research (VICI 91814650).

## Conflict of Interest

Author LB was employed by company JJP Biologics.

The remaining authors declare that the research was conducted in the absence of any commercial or financial relationships that could be construed as a potential conflict of interest.

## Publisher’s Note

All claims expressed in this article are solely those of the authors and do not necessarily represent those of their affiliated organizations, or those of the publisher, the editors and the reviewers. Any product that may be evaluated in this article, or claim that may be made by its manufacturer, is not guaranteed or endorsed by the publisher.
