# Supplementary material for: Anti-FcαRI Monoclonal Antibodies Resolve IgA Autoantibody-Mediated Disease
Source: Front Immunol. 2022 Mar 15;13:732977. doi: 10.3389/fimmu.2022.732977 (PMC8965572; doi:10.3389/fimmu.2022.732977)
Supplement: Supplementary file 1 [file Presentation_1.pptx]

## Slide 1
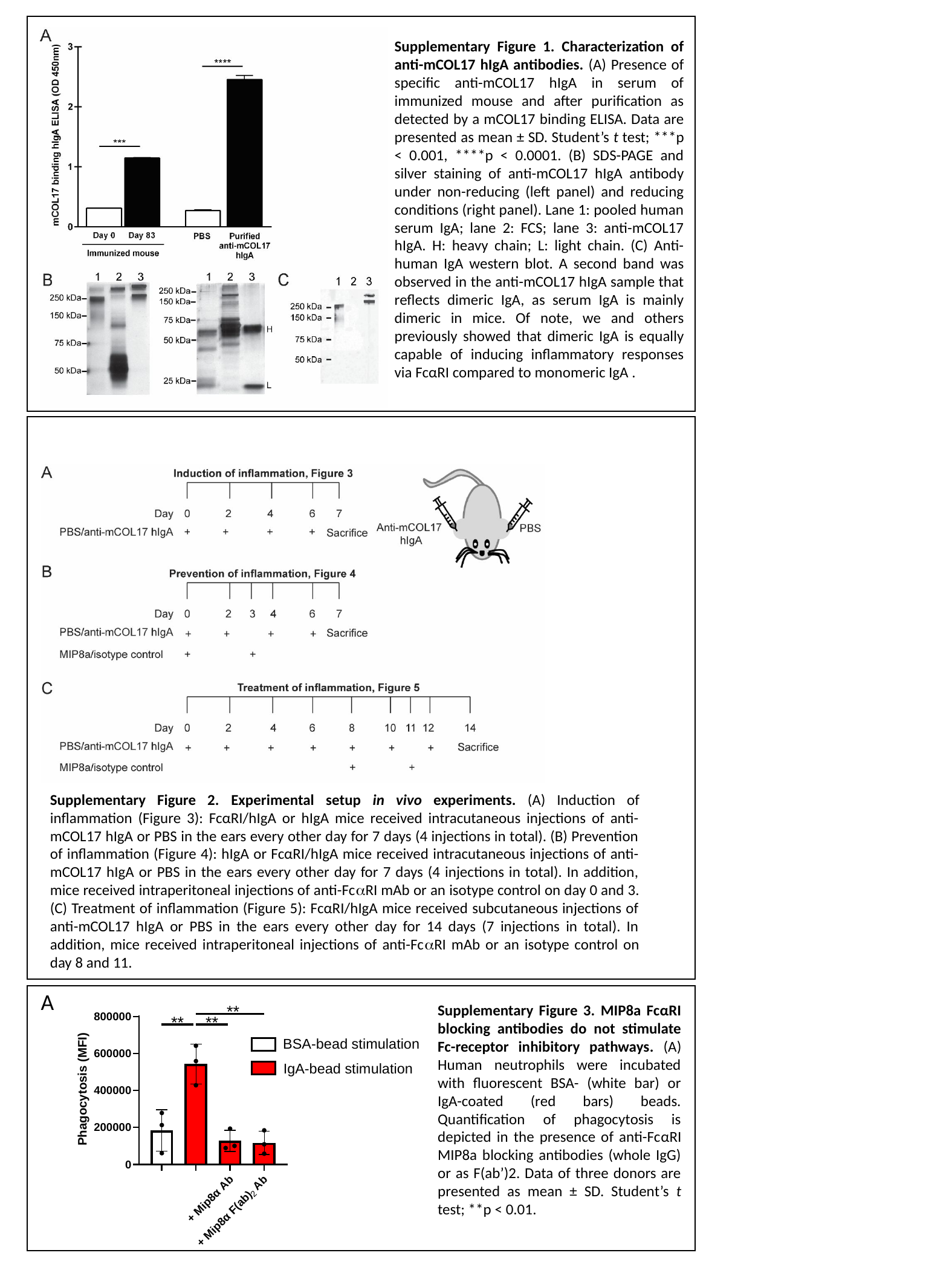

Supplementary Figure 1. Characterization of anti-mCOL17 hIgA antibodies. (A) Presence of specific anti-mCOL17 hIgA in serum of immunized mouse and after purification as detected by a mCOL17 binding ELISA. Data are presented as mean ± SD. Student’s t test; ***p < 0.001, ****p < 0.0001. (B) SDS-PAGE and silver staining of anti-mCOL17 hIgA antibody under non-reducing (left panel) and reducing conditions (right panel). Lane 1: pooled human serum IgA; lane 2: FCS; lane 3: anti-mCOL17 hIgA. H: heavy chain; L: light chain. (C) Anti-human IgA western blot. A second band was observed in the anti-mCOL17 hIgA sample that reflects dimeric IgA, as serum IgA is mainly dimeric in mice. Of note, we and others previously showed that dimeric IgA is equally capable of inducing inflammatory responses via FcαRI compared to monomeric IgA .
Supplementary Figure 2. Experimental setup in vivo experiments. (A) Induction of inflammation (Figure 3): FcαRI/hIgA or hIgA mice received intracutaneous injections of anti-mCOL17 hIgA or PBS in the ears every other day for 7 days (4 injections in total). (B) Prevention of inflammation (Figure 4): hIgA or FcαRI/hIgA mice received intracutaneous injections of anti-mCOL17 hIgA or PBS in the ears every other day for 7 days (4 injections in total). In addition, mice received intraperitoneal injections of anti-FcαRI mAb or an isotype control on day 0 and 3. (C) Treatment of inflammation (Figure 5): FcαRI/hIgA mice received subcutaneous injections of anti-mCOL17 hIgA or PBS in the ears every other day for 14 days (7 injections in total). In addition, mice received intraperitoneal injections of anti-FcαRI mAb or an isotype control on day 8 and 11.
A
Supplementary Figure 3. MIP8a FcαRI blocking antibodies do not stimulate Fc-receptor inhibitory pathways. (A) Human neutrophils were incubated with fluorescent BSA- (white bar) or IgA-coated (red bars) beads. Quantification of phagocytosis is depicted in the presence of anti-FcαRI MIP8a blocking antibodies (whole IgG) or as F(ab’)2. Data of three donors are presented as mean ± SD. Student’s t test; **p < 0.01.
